# Deletion of the K145R and DP148R Genes from the Virulent ASFV Georgia 2007/1 Isolate Delays the Onset, but Does Not Reduce Severity, of Clinical Signs in Infected Pigs

**DOI:** 10.3390/v13081473

**Published:** 2021-07-28

**Authors:** Anusyah Rathakrishnan, Ana L. Reis, Lynnette C. Goatley, Katy Moffat, Linda K. Dixon

**Affiliations:** The Pirbright Institute, Ash Road, Pirbright, Surrey GU24 0NF, UK; anusyah.rathakrishnan@pirbright.ac.uk (A.R.); ana.reis@pirbright.ac.uk (A.L.R.); lynnette.goatley@pirbright.ac.uk (L.C.G.); kathryn.moffat@pirbright.ac.uk (K.M.)

**Keywords:** African swine fever virus, macrophage, replication, virulence

## Abstract

African swine fever virus causes a frequently fatal disease of domestic pigs and wild boar that has a high economic impact across 3 continents. The large double-stranded DNA genome codes for approximately 160 proteins. Many of these have unknown functions and this hinders our understanding of the virus and host interactions. The purpose of the study was to evaluate the role of two virus proteins, K145R and DP148R, in virus replication in macrophages and virulence in pigs. To do this, the DP148R gene, alone or in combination with the K145R gene, was deleted from the virulent genotype II Georgia 2007/1 isolate. Neither of these deletions reduced the ability of the viruses to replicate in porcine macrophages compared to the parental wild-type virus. Pigs infected with GeorgiaΔDP148R developed clinical and post-mortem signs and high viremia, typical of acute African swine fever, and were culled on day 6 post-infection. The additional deletion of the K145R gene delayed the onset of clinical signs and viremia in pigs by 3 days, but pigs showed signs of acute African swine fever and were culled on days 10 or 13 post-infection. The results show that the deletion of DP148R did not attenuate the genotype II Georgia 2007/1 isolate, contrary to the results obtained with the genotype I Benin97/1 isolate. Additional deletion of the K145R gene delayed clinical signs, but infected pigs reached the humane endpoint. The deletion of additional genes would be required to attenuate the virus.

## 1. Introduction

The identification of genes encoding for virulence factors, and those inhibiting host immune responses, is key in generating rationally attenuated vaccines. This is especially important for the African swine fever virus (ASFV), for which no vaccine is available. ASFV is a large, complex double-stranded DNA virus, with a 170–193 kb genome that codes for at least 150 to 167 open reading frames (ORFs) [1,2]. Approximately 60 proteins have been identified in ASFV virions [3,4]. Although good progress has been made in determining the functions of ASFV proteins, many remain functionally uncharacterized [2].

ASFV causes African swine fever (ASF) disease in domestic pigs, wild boars and feral pigs. The spread of ASF into the Asian continent renders many rare and endangered pig species vulnerable to infection [5]. ASF can present as acute or chronic forms, depending on the infecting strain. Acute signs include high temperature, anorexia, skin and internal organ hemorrhaging, often leading to death. African wild suids, including warthogs and bush pigs, on the other hand, can be infected with only mild clinical signs and often without any signs, making them and *Ornithodoros* spp. soft tick vectors the reservoirs for ASFV [6].

ASFV gene functions have been elucidated via gene deletion or modification of the ASFV genome, using homologous recombination techniques that have been refined recently using CRISPR/Cas9 and single sorting methods [7,8,9,10,11,12]. Gene deletions from the virus genome can identify genes that are not essential for virus replication and help define the protein functions in cell cultures, and in vivo, in infected pigs [13,14,15]. The non-essential genes identified include many encoding proteins that inhibit host defense pathways, including type I interferon response, host antiviral pathways, apoptosis pathways and stress-induced responses [16,17]. Some of the gene deleted viruses have been shown to be attenuated in pigs and induced protection against challenge with a virulent virus. Thus, they show potential for use as candidates for live attenuated vaccines. For example, the deletion of I177L [18], different combinations of multigene family 360 or 505 genes, or the EP402R gene coding for the CD2v protein have identified candidate vaccine strains [19,20,21,22].

We previously demonstrated that deletion of the DP148R gene reduces the virulence of the genotype I strain Benin 97/1 and offers 100% protection against a homologous virulent isolate challenge [23]. In the current study, we deleted this gene from the genotype II isolate Georgia 207/1. We showed that deleting DP148R from this isolate did not attenuate the virus in pigs. We also tested the effect of deleting a second gene, K145R, from this virus. K145R is an abundant cytoplasmic protein that has previously been shown to be non-essential for the replication of ASFV in established cell lines. However, the effect of deleting this gene from a virulent isolate on virus attenuation in pigs has not previously been studied [24,25,26]. The encoded protein is immunogenic, and a potential negative serological marker for DIVA (differentiation of infected from vaccinated animals) diagnostic tests [4,27]. Deletion of the K145R gene from the Georgia∆DP148R virus did not affect growth in macrophages, it delayed the onset of clinical signs in infected pigs; however, pigs did not survive and were culled at a moderate severity humane endpoint.

## 2. Materials and Methods

### 2.1. Viruses and Cells

ASFV Georgia 2007/1, a virulent genotype II isolate, has been previously described [1]. Recombinant and wild-type viruses were propagated in porcine bone marrow cells (PBMs). PBMs were maintained in Earle’s Balanced Salt Solution (EBSS) (Thermo Fisher Scientific, Waltham, MA, USA) supplemented with 10% pig serum (Biosera, Nuaille, France) and 1% penicillin-streptomycin (Thermo Fisher Scientific, Waltham, MA, USA). Purified PBMs, without the presence of erythrocytes, were prepared by density gradient centrifugation using Histopaque-1083 (Sigma-Aldrich, Dorset, UK), at 1.083 g/mL. These cells were sustained with Roswell Park Memorial Institute (RPMI)-1640, supplemented with L-glutamine, 10% fetal bovine serum (FBS) (Life Science Production, Bedford, UK), 1% penicillin-streptomycin and 100 ng/ML porcine CSF-1 (Roslin Technologies, Midlothian, UK). Porcine alveolar macrophages were obtained from lung lavages, and maintained in RPMI supplemented with 10% pig serum and 1% penicillin-streptomycin solution. A wild boar cell line, WSL-R, was used in the first transfection step of manipulating the ASFV genome [8]. This was grown in a 1:1 ratio of Gibco^TM^ Ham’s F12 Nutrient Mix medium and Iscove’s Modified Dulbecco’s Medium (IMDM), supplemented with 10% FBS, 1% penicillin-streptomycin, and 1% L-Glutamine. Virus titrations were carried out by hemadsorption assays in PBMs, and titers were calculated using the Spearman and Kärber algorithm [28] and are expressed as HAD_50_/mL [29].

### 2.2. Recombinant Viruses

**Figure 1:**
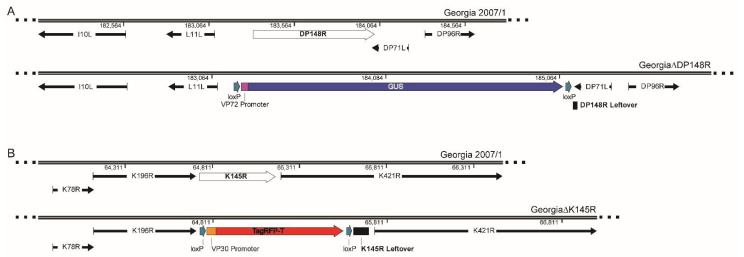
Schematic diagram depicting the deletion of DP148R and K145R. (**A**) ASFV Georgia 2007/1 genotype II strain (accession number: FR682468.1) is shown from position 182,064–184,800. The gene DP148R is deleted from position 183,187 to 184,012 and replaced with the reporter gene, GUS, driven by the ASFV P72 promoter. (**B**) ASFV Georgia 2007/1 is shown from position 63,811 to 66,473. The gene K145R is deleted from 64,734 to 65,086, leaving behind 85 bp of K145R on the 3′ end. This deletion is replaced by the fluorescent reporter, TagRFP-T driven by the ASFV VP30 promoter. Gene maps were designed using SnapGene^®^ Viewer software (GSL Biotech, San Diego, CA, USA) and modified on Adobe^®^ Illustrator^®^ CS6 (Adobe Systems Inc. San Jose, CA, USA).

Genetic modification of ASFV was ethically reviewed and carried out under license from the UK Health and Safety Executive. Two recombinant gene(s)-deleted ASFV, Georgia∆DP148R and Georgia∆K145R∆DP148R (Figure 1) were produced by homologous recombination between (i) transfer plasmids containing left and right flanking regions of the gene(s) to be deleted, and reporter genes under the control of ASFV promoters; and (ii) a parental virus [12,23].

The complete coding sequence of the DP148R gene was deleted and replaced with a β-Glucuronidase (β-GUS) reporter gene cassette [9]. To generate the transfer plasmid, p∆DP148R-VP72GUS, the flanking regions of DP148R were amplified by PCR and cloned upstream (535 bp) or downstream (601 bp) of the β-GUS gene cassette under the control of an ASFV VP72 promoter (Figure 1A). Porcine alveolar macrophages were infected with the ASFV Georgia 2007/1 isolate and then transfected with p∆DP148R-VP72GUS. Following homologous recombination between this plasmid and ASFV Georgia 2007/1 isolate, infected and transfected cells expressing the reporter GUS were detected via the addition of X-GLUC. The recombinant virus was purified in PBMs via multiple rounds of limiting dilutions.

The gene K145R was deleted and replaced by a fluorescent reporter gene, TagRFP-T [30]. The transfer plasmid, p∆K145R-VP30TagRFP-T, containing the upstream (587 bp) and downstream (536 bp) flanking arms of the K145R gene, and a TagRFP-T fluorescent marker under the control of ASFV VP30 promoter [31], was transfected into Georgia∆DP148R-infected WSL-R cells (Figure 1B). Using a single-cell isolation method via fluorescent-activated cell sorting (FACS), recombinant viruses expressing the red fluorescent protein gene were isolated and subsequently purified in PBMs, via a combination of single-cell isolation and limiting dilutions [12].

Viral DNA was extracted after the last round of single-cell isolation and after every round of limiting dilutions. The DNA was subjected to PCR amplification using internal primers, targeting the genes of interest in order to confirm the deletions and to verify the absence of the carry-over parental virus. The recombination site was then amplified and subjected to Sanger sequencing, to confirm the expected deletion and correct insertion of the reporter gene cassette.

### 2.3. Multistep Growth Curve

To measure growth over multiple rounds of infection, wild-type and recombinant gene(s)-deleted ASFV Georgia 2007/1 isolates were added to purified PBMs at a multiplicity of infection (MOI) of 0.01, in triplicate in 24-well plates. After an hour of incubation at 37 °C, the inoculum was removed and infected PBMs were washed once gently with Dulbecco’s phosphate-buffered saline (PBS), before adding fresh complete medium (RPMI 1640, supplemented with GlutaMAX™ (Gibco, Thermo Fisher Scientific, Waltham, MA, USA), 10% fetal bovine serum, 1% penicillin-streptomycin). Cells and supernatants were harvested every 24 h for 5 days and freeze-thawed thrice. The experiment was carried out in purified PBMs from 2 different pigs and all samples were titrated as described above. A repeated measures two-way ANOVA, where each row represented different days of infection, was used to evaluate the differences between the titers of different viruses recovered over time. Dunnett’s multiple comparison test was performed to evaluate the mean differences between recombinant viruses against the parental wild-type virus.

### 2.4. Pig Immunization Experiment

All animal experiments were conducted at the SAPO4 high containment animal housing at the Pirbright Institute (Woking, UK). Experiments were done in accordance with the regulated procedures from the Animals (Scientific Procedures) Act UK 1986 and conducted under Home Office License 7088520. All pigs were female Large White Landrace crosses, weighing between 15 and 23 kg. Titers of the virus inoculum were confirmed by back-titrations. Clinical signs, including temperatures, were recorded daily throughout the experiment and scored as described previously [32]. Pigs were terminated if they reached a moderate severity humane endpoint, as defined in the Home Office License. The scoring of gross lesions typical of ASFV infection was performed at necropsy [33]. With 4 pigs in a group, pigs were immunized intramuscularly with the two gene-deleted viruses, Georgia∆DP148R (Group A) and Georgia∆K145R∆DP148R (Group B).

### 2.5. Measurement of Viremia

Whole blood samples were collected in EDTA on different days during the experiments. The infectious virus in pig blood was titrated in PBMs, as described above, and the viremia is presented as HAD_50_/mL. The cut-off for accurate titers was 3.16 × 10^3^ HAD_50_/mL. Any value below this cut-off is considered to be the detection of infectious ASFV, but the titers may not be precise. A repeated measures two-way ANOVA, with Sidak’s multiple comparison test, where each row represented days post-infection, was used to evaluate the mean differences in the levels of infectious viruses detected in blood over time, using GraphPad Prism version 7.00 for Windows (GraphPad Software, La Jolla, CA, USA).

### 2.6. ELISA to Detect ASFV-Specific Antibodies

The antibody responses of Group B to the ASFV VP72 protein were measured on different days post-immunization using a blocking ELISA (INgezim PPA COMPAC, Ingenasa, Madrid, Spain), as described by the manufacturer. The final optical density (OD) was read at 450 nm on a microplate reader BioTek, with Gen5 software (Winooski, VT, USA). The percentage of blocking was calculated using the following equation: (negative control OD—sample OD)/(negative control OD—positive control OD) × 100%. Samples above 50% blocking were considered to be positive, while anything below 40% was considered to be negative. Samples with blocking between 40–50% were considered to be doubtful.

### 2.7. IFN-γ ELISpot Assay

Peripheral blood mononuclear cells (PBMC), were collected from all pigs before immunization, and from 2 pigs on day 13. PBMC were isolated from whole blood collected in EDTA, using density gradient centrifugation with Histopaque-1083 (Sigma-Aldrich, Dorset, UK). Multiscreen-IP filter plates with 0.45 μm pore size (MAIPS4510, Millipore, Burlington, MA, USA) were coated with 4 μg/mL capture anti-IFN-γ (P2F6, Thermo Fisher Scientific, Waltham, MA, USA) in 0.05 M carbonate-bicarbonate coating buffer, and stored overnight at 4 °C. After washing the plates, pig PBMC were seeded in RPMI-1640, Glutamax, supplemented with 10% FBS, 1% of penicillin-streptomycin (10,000 U/mL) and 50 μM β-mercaptoethanol. PBMC were stimulated with Georgia 2007/1 ASFV at 10^6^ HAD_50_/mL, an equivalent amount of mock-infected inoculum as negative control and PHA at 20 μg/mL, as a positive control. PBMC were incubated at 37 °C for 16–18 h, and then lysed by incubation in water for 5 m, followed by PBS washes. Next, 1 μg/mL biotinylated anti-IFN-γ monoclonal antibody (P2C11, Thermo Fisher Scientific, Waltham, MA, USA) was added, and the plates were incubated at room temperature for 2 h and then washed with PBS. Diluted streptavidin alkaline phosphatase conjugate (Invitrogen, Thermo Fisher Scientific, Waltham, MA, USA) was added, and the plates were further incubated for an hour at room temperature. Lastly, alkaline phosphatase substrate (Bio-Rad, Watford, UK) was added to develop the spots. Spot-forming cells (SFC) were counted on an ELISpot reader (ImmunoSpot, CTL, Bonn, Germany). A repeated measures two-way ANOVA, with Sidak’s multiple comparison test, where each row represented days post-infection, was used to evaluate the mean differences in the number of IFN-γ-producing cells over time using GraphPad Prism version 7.00 for Windows (GraphPad Software, La Jolla, CA, USA).

## 3. Results

### 3.1. Recombinant Gene-Deleted Viruses

The DP148R gene from the Georgia 2007/1 genotype II strain (accession number: FR682468.1 isolate) from genome position 183,187 to 184,012 was deleted using a similar strategy as described previously for genotype I Benin 97/1 [23] to produce Georgia∆DP148R. The complete coding region of DP148R was deleted, apart from a 25 bp sequence at the 3′ end, which overlaps with the 3′ end of the adjacent downstream gene, DP71L [23] (Figure 1A).

The K145R gene was selected for deletion since it is known to be a non-essential gene and is immunogenic [24,25,27]. Viruses with this gene deleted may therefore be candidates for diagnosis to differentiate infected from vaccinated animals (DIVA compliant vaccines). The K145R gene was deleted from the ASFV Georgia genome (position 64,734 to 65,086), apart from 85 bp at the 3′ terminal that may contain the promoter for the adjacent downstream K421R gene (Figure 1B). This gene was deleted from the recombinant Georgia∆DP148R isolate to produce a double gene-deleted virus Georgia∆K145R∆DP148R.

### 3.2. Replication of Recombinant Gene Deleted ASFV in Primary Porcine Bone Marrow Cells

**Figure 2:**
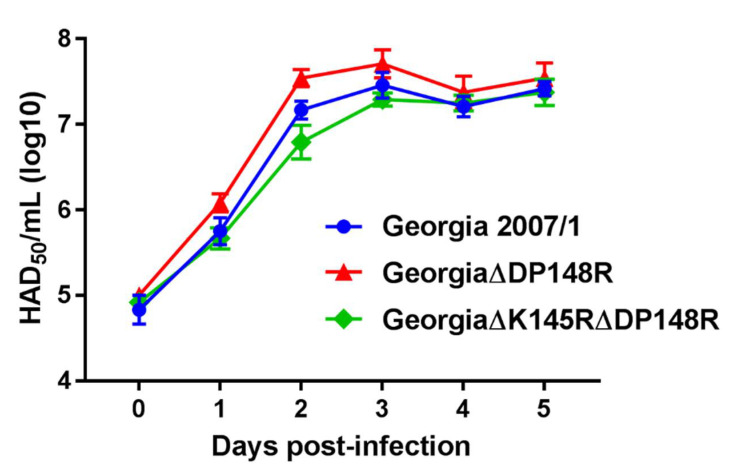
Replication of the recombinant ASFV viruses compared to the wild-type Georgia 2007/1 strain. Purified PBMs from 2 different pigs were infected with viruses at MOI 0.01 in triplicates. Viruses were harvested from both cells and supernatants at different time points and titrated on PBMs in quadruplicates. Day 0 represents the inoculum. A two-way ANOVA with Dunnett’s multiple comparisons test was performed, and no differences were observed between the recombinant virus and wild-type Georgia 2007/1 isolate.

The replication kinetics of recombinant ASFVs in porcine macrophages were compared to the virulent Georgia 2007/1 isolate (Figure 2). PBMs from 2 different pigs were infected at a low multiplicity of infection (0.01) and viruses from both cells and supernatant were harvested together every day post-infection for 5 days, and the yields of the infectious virus were titrated. The recombinant Georgia∆DP148R and Georgia∆K145R∆DP148R viruses showed similar growth kinetics and titers to the Georgia 2007/1 isolate, over the five days of culture (Figure 2).

### 3.3. Infection of Pigs with Georgia∆DP148R or Georgia∆K145R∆DP148R

**Figure 3:**
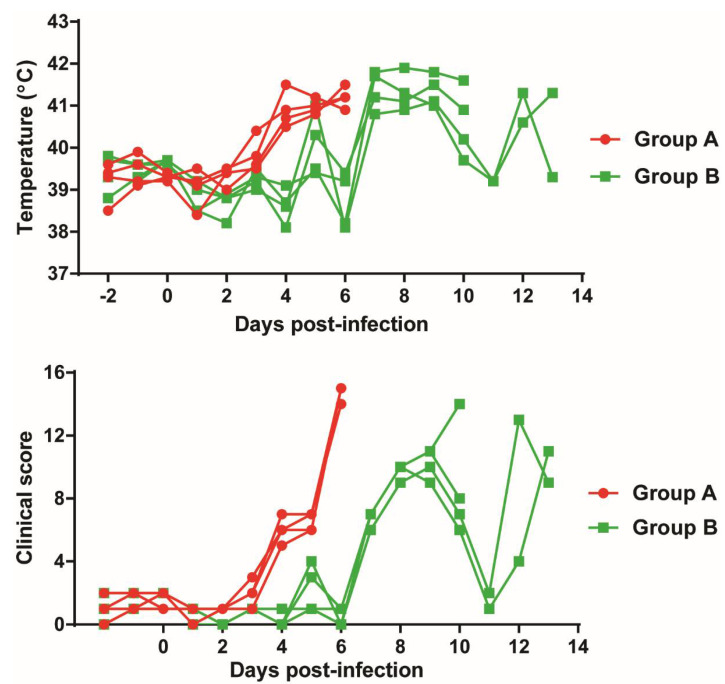
Temperatures and clinical scores. Pigs were immunized with either Georgia∆DP148R (Group A) or Georgia∆K145R∆DP148R (Group B). (**A**) Line graphs show the daily rectal temperatures of infected pigs. (**B**) Cumulative clinical scores, based on clinical signs, observed daily, are shown.

Two groups of four pigs were immunized intramuscularly (IM) with 1 mL of 10^3^ HAD_50_ Georgia∆DP148R (Group A) or Georgia∆K145R∆DP148R (Group B). Rectal temperatures and clinical scores were recorded daily for all pigs [32]. All 4 pigs in Group A showed typical signs of acute ASFV, including an increase in temperature above 40.5 °C, starting at 4 days post-infection (dpi), followed by lethargy and anorexia, and temperatures above 41 °C (Figure 3). By 6 dpi, all 4 pigs reached a moderate severity humane endpoint and were euthanized.

In group B, the increase in temperature in pigs immunized with Georgia∆K145R∆DP148R was delayed. Pig B4 had a temperature above 40.5 °C at 5 dpi, which then reduced at 6 dpi. At 7 dpi, all pigs developed a fever above 40.5 °C, and showed reduced appetite at 8 and 9 dpi (Figure 3). Pigs B1 and B2 were euthanized on 10 dpi at the humane endpoint. The other pigs (B3 and B4) showed a transient decrease in temperature at 11 dpi, but this increased again, and both were euthanized at 13 dpi at the humane endpoint (Figure 3). Thus, the double deletion of K145R and DP148R genes delayed the onset of clinical signs by 2 or 3 days, relative to the single DP148R deletion, but all the pigs were euthanized at the moderate severity humane endpoint.

**Figure 4:**
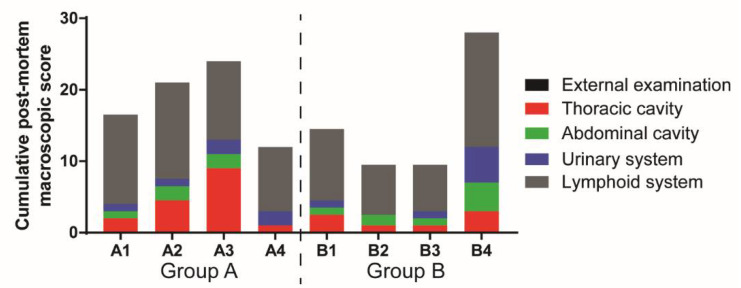
Post-mortem macroscopic lesion scoring. Lesions observed were scored and displayed as a cumulative score, observed in different organs of the pig. The external examination included general body condition and lesions in eyes, conjunctiva, nostrils, oral cavity, skin, subcutis, skeletal muscles and joints (black bar). Lesions in the thoracic cavity (red bar) include the presence of thoracic exudates, as well as lesions affecting the cardio-respiratory system. Lesions in the abdominal cavity (green bar) include the presence of ascites, along with the presence of lesions affecting the gastrointestinal system, including the stomach, intestines, liver and gallbladder. Lesions observed in the urinary system (kidneys and urinary bladder) are included in the blue bar. Finally, lesions depicted by the grey bar include pathology observed in lymphoid tissues: tonsils, thymus, spleen and various lymph nodes.

Necropsies of Groups A and B pigs showed lesions characteristic of acute ASF infections (Figure 4). In Group A, these included erythematous tonsils, hydropericardium, ascites, enlarged spleen, enlarged and edematous lymph nodes with hemorrhages, sometimes resembling blood clots (submandibular, retropharyngeal, tracheobronchial, gastrohepatic, renal and mesenteric lymph nodes). Two pigs also displayed enlarged hearts, with petechial hemorrhages in the epicardium (A3 and A4), mild to moderate lack of lung collapse with rib impressions (A1, A2—mild; A3—moderate), a variable degree of congestion or hyperemia in the lungs (A2 and A3) and the moderate presence of multifocal petechiae in the renal cortex (A3 and A4). Except for pig B4, group B pigs had relatively lower cumulative lesion scores (9.5–14.5) compared to Group A (12–24) (Figure 4). Pig B4 had additional gross lesions compared to the other group B pigs. These included the presence of petechial hemorrhages in the epicardium, hepatomegaly with the presence of multifocal punctiform hemorrhages, as well as petechial hemorrhages in the gallbladder and bladder. Pig B4 did not show changes in spleen size, although all observed lymph nodes were enlarged and displayed hemorrhages of different extensions and severity.

### 3.4. Levels of Virus in the Blood

**Figure 5:**
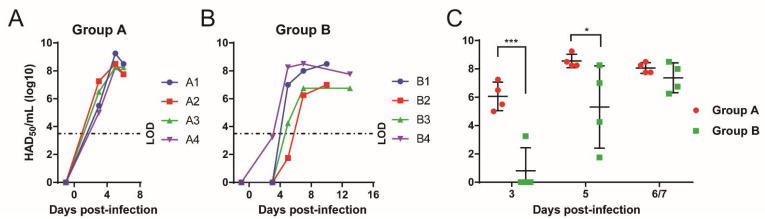
Viremia levels of pigs. Levels of infectious virus were measured for pigs immunized with (**A**) Georgia∆DP148R and (**B**) Georgia∆K145R∆DP148R via titrations of blood after inoculation. The limit of detection (LOD) using this method is 3.16 × 10^3^ HAD_50_/mL. (**C**) Comparison of the infectious virus levels between Group A and B at the early days of infection. A two-way ANOVA with Sidak’s multiple comparisons test was performed, to evaluate the differences in the levels of viruses over days of infection. Significant difference is represented by asterisk(s); differences are represented by asterisk(s) where * is *p* < 0.05 and *** is *p* < 0.001.

Levels of infectious virus in the whole blood of pigs from Groups A and B were determined by titration on PBMs (Figure 5). In group A, viremia was detected from 3 dpi onwards (10^5^ to 10^7^ HAD_50_/mL), and continued to increase at 5 dpi (10^8^ to 10^9^ HAD_50_/mL) (Figure 5A). In comparison, only pig B4 had detectable virus in the blood at 10^3^ HAD_50_/mL on 3 dpi (Figure 5B). On 5 dpi, pigs B1 and B4 had high levels of viremia (10^7^ and 10^8^ HAD_50_/mL, respectively) while pigs B2 and B3 had the virus detected at 10^1^ and 10^4^ HAD_50_/mL. By day 7, all 4 pigs had viremias, ranging from 10^6^ to 10^8^ HAD_50_/mL. At termination on 10 dpi, pigs B1 and B2 had 10^8^ and 10^7^ HAD_50_/mL blood, while at 13 dpi, pigs B3 and B4, had 10^6^ and 10^7^ HAD_50_/mL, respectively (Figure 5B). Thus, pigs in group B had a significant delayed onset of viremia and lower peak levels of viremia compared to pigs in Group A at 3 (*p* = 0.0003) and 5 dpi (*p* = 0.0202) (Figure 5C). This shows that the additional deletion of K145R had a small attenuating effect on Georgia∆DP148R.

### 3.5. Early Indication of Immune Response against ASFV

**Figure 6:**
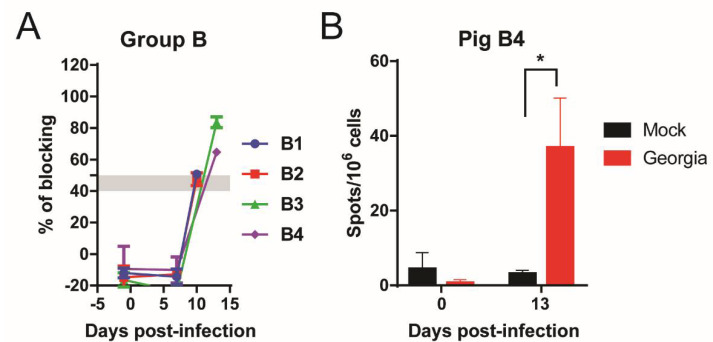
Early indications of host immune response. (**A**) Antibody responses of pigs immunized with Georgia∆K145R∆DP148R (Group B) were measured on different days after immunization, using a blocking ELISA, against the ASFV VP72 protein. Results are presented as the percentage of blocking, where values above 50% blocking were considered as a positive antibody response, while anything below 40% was considered to be negative. Samples with blocking between 40–50% were considered to be doubtful. (**B**) The IFN-γ producing cells levels of pig B4 (immunized with Georgia∆K145R∆DP148R) at its termination endpoint were measured via ELISpot, by stimulating its PBMC with Georgia 2007/1 isolate. Results are presented as mean frequencies IFN-γ producing cells per million PBMC. A two-way ANOVA with Sidak’s multiple comparisons test was performed to evaluate the differences between mock- and Georgia 2007/1-stimulated cells. The significant difference is represented by an asterisk (*), where *p* < 0.05.

Pig antibody responses to ASFV p72 protein were measured via a blocking ELISA assay using serum extracted at different time points after infection. Despite being culled early in the experiment, pigs from Group B had mounted antibody responses (B1, B2: doubtful, 10 dpi; B3 and B4: positive at 13 dpi) (Figure 6A).

PBMC were isolated from the blood of pigs B3 and B4 at the termination endpoint (13 dpi) and were kept frozen in 90% FBS and 10% DMSO. These frozen PBMC were subsequently used in IFN-γ ELISpot assay, to evaluate the number of IFN-γ producing cells when stimulated with ASFV Georgia 2007/1 isolate, if any. PBMC from pig B3 did not respond to the positive control, PHA, and were discarded. However, for pig B4, a significant increase in the number of IFN-γ producing cells was noted at 13 dpi compared to before immunization (*p* = 0.0171) (Figure 6B).

## 4. Discussion

The spread of ASF, now on three different continents, has had a severe socio-economic impact in affected countries, with a large effect globally on pork prices and supplies. The development of an ASF vaccine is impeded by the limited information available on the functions of many ASFV-encoded proteins during the infection of porcine macrophages or the infection of pigs. This also restricts our understanding of virus–host interactions that are important for cellular tropism, pathogenesis, and the induction of protection against challenge. Here we investigated the impact of deleting two different genes, DP148R and K145R, from the virulent genotype II ASFV Georgia 2007/1 isolate.

DP148R, an early expressed gene of ASFV, was previously deleted from a genotype I isolate (Benin 97/1), without any effect on viral growth in macrophages [23]. When tested in pigs, despite moderate clinical signs and viremia after immunization, pigs were afforded 100% protection against challenge with the homologous virulent isolate [23]. Here, we made the same gene deletion in the genotype II, Georgia 2007/1, isolate. This gene-deleted virus had similar growth kinetics to the wild-type virus in macrophages in vitro, but was not attenuated when tested in pigs, as evidenced by the increase in temperatures and development of clinical signs as early as day 3 post-infection. All four pigs infected with Georgia∆DP148R reached their humane endpoint at 6 days post-infection, as was observed in infections with the wild-type virulent virus, e.g., the Georgia 2007/1 isolate [34]. Similar results were shown for the deletion of the DP148R gene from a Chinese genotype II isolate (HLJ/18), where pigs that were immunized intramuscularly died within 9 days of infection [21]. The DP148R protein is well conserved in virulent ASFV isolates. The sequence of DP148R is 98% identical between Benin 97/1 and Georgia 2007/1 strains; therefore, we assume that the protein has the same function. Our unpublished observations indicate that DP148R has a role in inhibiting type I IFN responses. ASFV codes for a number of proteins that inhibit type I IFN responses, and deletion of the genes for these can reduce the virus virulence. We, therefore, suggest that the different results we obtained from the deletion of DP148R from the Georgia 2007/1 compared to Benin 97/1 isolates may result from variations in the presence or sequence of genes for other virulence factors encoded by their genomes [1].

The K145R gene is transcribed to high levels and encodes a protein with high amino acid identity between different genotypes. The protein is expressed late during the infection of cells [25,35], and is an abundant cytoplasmic protein [4,36]. However, K145R was not detected in virus particles [25]. A possible role for K145R as an inhibitor of stress-induced apoptosis has been suggested [24]. This protein was one of the 14 highly immunogenic proteins identified using a lambda phage BA71V cDNA expression library and serum from Malta-infected domestic pigs [27]. In addition, sera from several pigs convalescing from ASFV infections reacted with cells that were transiently expressing the K145R protein [25]. Hence, the data suggest that K145R is an immunogenic protein, and deletion of the gene could possibly serve as a DIVA vaccine marker, since antibodies against K145R would be detected in infected animals but not in vaccinated animals.

With this in mind, we deleted K145R from Georgia∆DP148R to produce a double gene-deleted Georgia virus, Georgia∆DP148R∆K145R. The deletion of K145R from Georgia∆DP148R did not affect viral growth kinetics in macrophages, in agreement with previous results following the deletion of the K145R gene from two ASFV isolates, Ba71V [24] and Kenya 1033 [26]. In contrast, a slightly delayed replication was noted when K145R was deleted from the Armenia strain, although it was uncertain if this delay was caused by the K145R deletion, or whether it was due to the insertion of the vaccinia thymidine kinase transgene in the construct [25].

The additional deletion of K145R, alongside DP148R, from the Georgia isolate caused a delay of 3 days in the development of clinical signs in infected pigs, compared to pigs infected with the Georgia∆DP148R virus. Interestingly, the temperatures and clinical scores seemed to drop, notably in pigs B3 and B4, from day 10 to 11, an indication of perhaps an attempt by the host to control the infection. This was further supported by the presence of VP72 antibodies in pigs B3 and B4, and the presence of IFN-γ-producing cells in pig B4 at day 13 post-infection. Viremia was clearly delayed in pigs infected with Georgia∆DP148R∆K145R, compared to the single deletion of DP148R. However, the levels of infectious virus subsequently reached high levels and did not recede over time. All pigs infected with Georgia∆DP148R∆K145R reached the moderate severity humane endpoint and were culled by 13 days post-inoculation. This shows that the deletion of K145R had attenuated the Georgia∆DP148R virus, but this was not sufficient to avoid reaching the moderate severity endpoint. We did not test the impact on virus attenuation of deleting the K145R gene alone, so we cannot exclude the possibility of synergy between the functions of the two genes in virus attenuation.

Since the K145R gene codes for an abundant cytoplasmic immunogenic protein and can be deleted to partially attenuate a virulent ASFV isolate, this makes K145R a potentially important negative serological target for the development of diagnostic tests to distinguish infected from vaccinated animals (DIVA). The use of a vaccine with DIVA potential would be an advantage for monitoring vaccination and for eventual confirmation of freedom from the disease. The deletion of additional genes to attenuate the virus, including DP148R, will be necessary to fine-tune the levels of virus attenuation. A combination of several gene deletions would also improve the safety of the vaccine, by reducing the chances of a reversion to virulence during its passage in pigs.

## Data Availability

Not applicable.
